# The Effect of a Compound Protein on Wound Healing and Nutritional Status

**DOI:** 10.1155/2022/4231516

**Published:** 2022-03-24

**Authors:** Xue Wang, Zhangping Yu, Shengnan Zhou, Shiwei Shen, Wei Chen

**Affiliations:** ^1^Department of Clinical Nutrition, Peking Union Medical College Hospital, Chinese Academy of Medical Sciences and Peking Union Medical College, No. 1, Shuaifuyuan, Wangfujing, Dongcheng District, Beijing 100730, China; ^2^Department of Endocrinology, The Affiliated Wuxi No. 2 Hospital of Nanjing Medical University, 68 Zhongshan Road, Wuxi, Jiangsu 214002, China

## Abstract

Proteins provide the main building blocks for tissue growth, cell renewal, and repair during wound healing. We aimed to examine the effect of a compound protein on wound healing, nutritional status, and underlying mechanisms. We first performed a preliminary experiment to identify the appropriate wound healing assessment points. In the formal experiment, there were five groups (control group: 8.3750 g/kg/day saline solution; model group: 8.3750 g/kg/day saline solution; whey protein group: 8.3750 g/kg/day whey protein; low-dose compound protein group: 4.1875 g/kg/day compound protein; and high-dose compound protein group: 8.3750 g/kg/day compound protein) with eight rats in each group. At each turning point, we observed the wound healing rate and nutritional status of the different groups of rats. In addition, biochemical assays were used to determine the mechanisms underlying the effects of the compound protein. In the preliminary experiment, the third day after modeling was the turning point between the inflammatory and proliferation phases, and the eighth day was the turning point between the proliferation and remodeling phases. The formal experiment evaluated wound healing condition, inflammatory response, angiogenesis, collagen deposition, and nutritional status. A pathological report showed increased vascularization, collagen deposition, and epithelialization in compound protein-treated groups. Protein-treated mice showed decreased interleukin (IL)-6, IL-8, neutrophils, and lymphocytes and increased IL-10, albumin, prealbumin, total protein levels, insulin-like growth factor 1 (IGF-1), fibroblast growth factor 2 (FGF-2), and vascular endothelial growth factor (VEGF) expressions. All parameters were significant (*p* < 0.05) compared to the model group. There was a dose-dependent effect of the compound protein. The accelerated wound healing mechanism may be that the compound protein accelerates the whole wound healing process, making wounds transition from the inflammatory phase to the proliferation phase faster, entering the remodeling phase earlier. Administration of a compound protein can accelerate wound healing and improve the nutritional status.

## 1. Introduction

For many surgery or trauma patients, an increased wound healing time results in poor recovery and increases the burden on patients and public health. Studies have found that several factors influence metabolism, such as the stress of surgery, fasting before the operation, and wound infection [1]. Rapid wound healing is characterized by accelerated protein, glycogen, and fat catabolism [2]. Moreover, malnutrition is a major risk factor for postoperative complications [3] and can influence patient outcomes such as morbidity, mortality, length of stay, inflection rates, and wound healing [4, 5].

The wound healing process has traditionally been divided into three phases: inflammation, proliferation, and remodeling [6]. A myriad of orchestrated reactions and interactions between cells and chemicals occur within each phase. In addition, the phases overlap considerably. However, specific events occur in each phase. The major traits of the hemostasis and inflammation phases are chemotaxis, activation-vasodilation, cytokine release, and inflammatory cell gathering. During the proliferation phase, epithelization, angiogenesis, and provisional matrix formation occur. The main feature of the remodeling phase is collagen deposition in an organized and well-mannered network [7]. Proteins provide the main building blocks for tissue growth, cell renewal, and repair throughout the wound healing process. Proteins significantly affect the entire process of wound healing through their roles in RNA and DNA synthesis, collagen and elastic tissue formation, immune system function, epidermal growth, and keratinization [8]. Therefore, it is vital to provide proteins for wound healing.

Two forms of proteins are absorbed through the intestinal tract: amino acids and oligopeptides [9, 10]. When a protein enters the intestinal tract, it is first metabolized into amino acids and oligopeptides and then assimilated in the form of amino acids or oligopeptides. Studies have demonstrated that the total absorption of amino nitrogen is greater during perfusion of solutions containing partial enzymatic hydrolysates of protein (consisting mainly of peptides with 2–6 amino acid residues) than during intestinal perfusion of the corresponding free amino acid mixtures with identical amino acid compositions [11, 12]. In this study, we used an existing product composed of a different source of nitrogen (whey protein and some functional oligopeptides) and called it a compound protein. The aims of this study were 1) to test whether the administration of oligopeptides can accelerate wound healing and improve nutritional status and 2) to identify the potential mechanism of these oligopeptides and whether the compound protein affects wound healing.

## 2. Materials and Methods

### 2.1. Animals and Groups

This study was approved by the Animal Ethical Committee of Peking Union Medical College Hospital, China (number: XHDW-2017-020), and the experimental procedures were performed in strict compliance with the Guide for the Care and Use of Medical Laboratory Animals (Ministry of Health, China, 1998). Furthermore, efforts were made to minimize pain in the animals.

Male SD rats (weighing 200–250 g, Cavens, China) were housed under standard room temperature and humidity conditions with a 12/12 h light/dark cycle with free access to water and food. The rats were acclimatized for one week before experimentation.

Before the formal experiments, we determined the turning points between the inflammation and proliferation phases and the proliferation and remodeling phases. We divided the four rats into two groups (high-dose compound protein and control groups), and the high-dose compound protein group rats were administered the compound protein (8.3750 g/kg/day) by gavage. The control group rats were administered the same volume of the saline solution via gavage. In the formal experiment, there were five groups (control, model, whey protein, low-dose compound protein, and high-dose compound protein groups), with eight rats in each group. In the low-dose compound protein group, rats were administered a low dose of the compound protein (4.1875 g/kg/day). In the high-dose compound protein group, rats were administered a high dose of the compound protein (8.3750 g/kg/day). In the whey protein group, the rats were administered whey protein (8.3750 g/kg/day, Nutrasumma, 5.89 g whey protein/10 g). In the control and model groups, the rats were administered the same volume of the saline solution (8.3750 g/kg/day).

### 2.2. Composition of the Compound Protein

The amino acid composition of the compound protein is listed in Table 1.

### 2.3. Model

Rats were anesthetized with 40 mg/kg pentobarbital administered intraperitoneally, the hair on the back of the rats was removed, and the area was sterilized. Two round wounds (diameter, 2.5 cm) were made on the backs of the rats, and the skin was removed deep into the subcutaneous tissue. Sterile dressings were used to stop the bleeding, and a wound model was established. Each rat was used as a wound model, except for the rats in the control group. After modeling, each rat was housed in a single cage to prevent wounds from being bitten by others. Blood and tissue samples around the wounds were collected from all rats at two specific turning points. Blood samples were centrifuged and stored at −80°C in a refrigerator (Haier).

### 2.4. Rate of Wound Healing

After modeling, the wounds were observed every day, and the shapes of the wounds were traced on semitransparent paper every two days until the wounds healed completely. Photos of the paper were taken with a camera (Canon, 800D) at a fixed distance (15 cm), and these photos were uploaded (Photoshop CS5) to calculate the area of the wounds. Finally, we calculated the rate of wound healing after modeling to analyze the status of wound healing [13].

(1) $$\begin{array}{c} \text{rate of wound healing}=\frac{\text{initial wound area}-\text{nonhealing wound area}}{\text{initial wound area}}*100. \end{array}$$

### 2.5. ELISA

The serum levels of total protein (TP), albumin (ALB), and prealbumin (PA) were measured using ELISA kits (Nanjing Jiancheng, China). The levels of inflammatory factors IL-6 (Multi Sciences, China), IL-8 (Sbjbio Scientific, Inc., China), and IL-10 (Multi Sciences, China) were also quantified using ELISA kits. All the assays were performed using a microplate reader (Thermo Fisher Scientific). The hydroxyproline concentration in the tissues around the wounds was determined using a hydroxyproline assay kit (Nanjing Jiancheng, China). The ELISA was performed according to the manufacturer's instructions.

### 2.6. Blood Cell Count

All blood samples were assessed using an automatic blood cell analyzer (Nihon Kohden) to count the neutrophils and lymphocytes.

### 2.7. Histology

We cut tissues around the wounds at the turning points for histological analysis. The tissues were fixed with a formalin solution, and the formalin-fixed tissues were embedded in paraffin and sectioned. Tissues obtained from rats at various time points and from different groups were stained with H&E and the Masson trichrome. Images of the sections were captured using a microscope (Olympus).

### 2.8. mRNA Analysis

RNA from rats was isolated using a kit (TransGen Biotech, China) and TRIzol reagent (TransGen Biotech, China), according to the manufacturer's instructions. Reverse transcription was performed using a Reverse Transcription Kit (TransGen Biotech). Quantitative real-time PCR was performed for insulin-like growth factor 1 (IGF-1), fibroblast growth factor 2 (FGF-2), and vascular endothelial growth factor (VEGF), using specific primers (Sangon Biotech, China) on a Mastercycler EP Realplex (Eppendorf).

### 2.9. Statistical Analysis

Wound healing rates were analyzed using two-way ANOVA. Other results were analyzed using one-way ANOVA, and multiple comparisons were performed using the least significant difference (LSD) method. Differences were considered significant if the probability of the difference occurring by chance was less than 5 in 100 (*p* < 0.05).

## 3. Results

### 3.1. Turning Points

During the pilot experiment, we observed the wound healing rate daily. Based on the H&E staining results of the rats in the control group, on the third day after modeling, fibroblast proliferation, angiogenesis, and epidermization appeared in the tissues around the wounds, indicating the proliferation phase. In contrast, on the eighth day after modeling, considerable collagen was observed in the tissues around the wounds, indicating that the tissues were in the remodeling phase. Based on these findings, we regarded the third day after modeling as the turning point between the inflammation and proliferation phases and regarded the eighth day as the turning point between proliferation and remodeling phases. Considering the above observations, we decided to collect blood samples and tissues on the third and eighth days after modeling, respectively.

**Figure 1:**
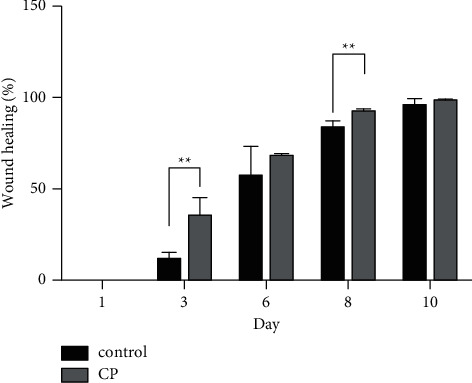
Rate of wound healing in two groups. Control indicates the control group (*n* = 2); CP indicates the compound protein group (*n* = 2). ^*∗∗*^indicates that the significance between the control and compound protein groups was less than 0.01 (*p* < 0.01).

Based on the wound healing rate (Figure 1), we found that the rate of wound healing increased over time. However, the rate of wound healing in the compound protein group was significantly higher than that in the control group on the third and eighth days after modeling, which demonstrated that providing the compound protein could increase the wound healing rate in addition to the basic energy intake.

### 3.2. Rate of Wound Healing

**Figure 2:**
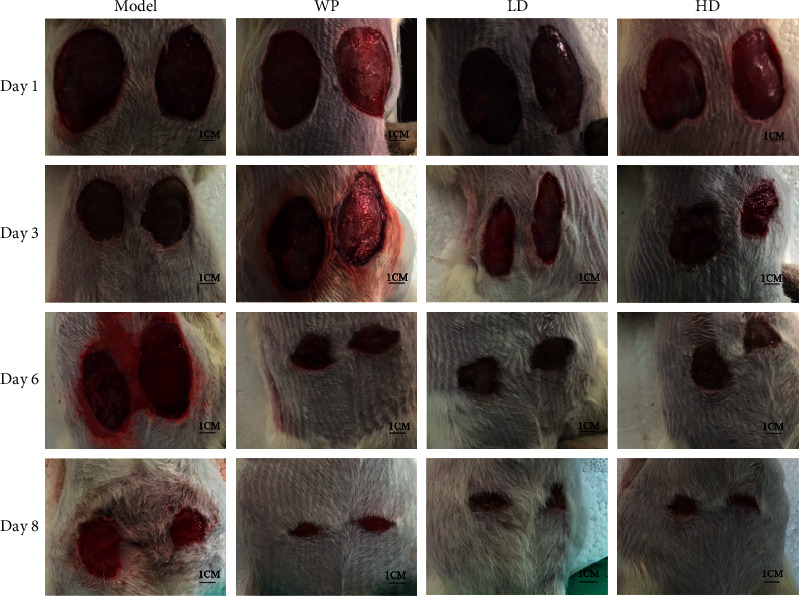
Change in the wounds of rats in different groups after modeling during the wound healing process. Model indicates the model group; WP indicates the whey protein group; LD indicates the low-dose compound protein group; HD indicates the high-dose compound protein group.

**Figure 3:**
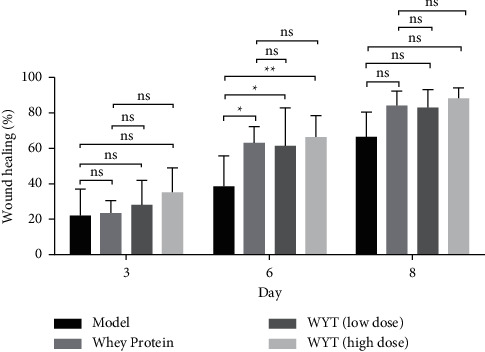
Rate of wound healing in the different groups. Model indicates the model group; WP indicates the whey protein group; LD indicates the low-dose compound protein group; HD indicates the high-dose compound protein group. ^*∗*^indicates that the significance between two groups was less than 0.05 (*p* < 0.05); ^*∗∗*^indicates that the significance between two groups was less than 0.01 (*p* < 0.01).

Figure 2 shows the changes in wounds during the wound healing process after modeling in each group. The area of the wounds on the rats in each group decreased over time, but the area decreased faster in the whey protein, low-dose compound protein, and high-dose compound protein groups than in the model group.  Figure 3 shows the quantitative analysis of wound healing, the rate of wound healing of the four groups on the third day, sixth day, and eighth day after modeling. The rate of wound healing in the three treatment groups was higher than that in the model group. The rate in the high-dose compound protein group was higher than that in the whey protein group. Moreover, the rates in the whey protein, low-dose compound protein, and high-dose compound protein groups were significantly different from those in the model group on the sixth day (*p* < 0.05).

### 3.3. Evaluation of Plasma Protein Markers

**Figure 4:**
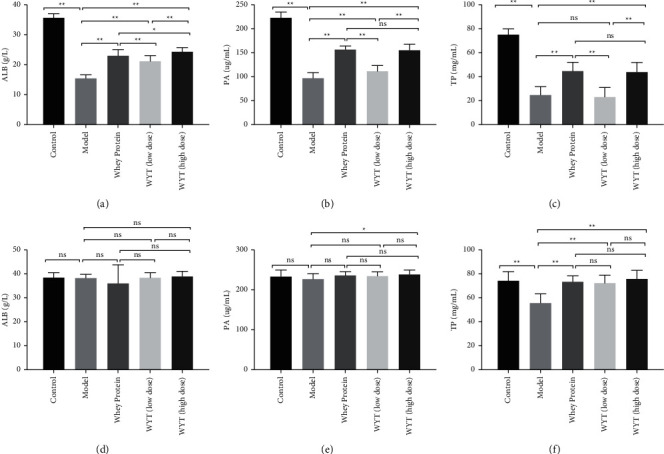
The plasma protein level of rats on the third day (a–c) and the eighth day (d–f) after modeling. ALB indicates albumin; PA indicates prealbumin; TP indicates total protein. Model indicates the model group; WP indicates the whey protein group; LD indicates the low-dose compound protein group; HD indicates the high-dose compound protein group. ns indicates that the significance between the two groups was not significant. ^*∗*^indicates that the significance between two groups was less than 0.05 (*p* < 0.05); ^*∗∗*^indicates that the significance between two groups was less than 0.01 (*p* < 0.01).

In this study, we measured the levels of three protein markers in rat serum: albumin (ALB), prealbumin (PA), and total protein (TP). ALB, PA, and TP levels were used as indicators of nutritional status.  Figure 4 depicts the changes in these markers after modeling. We found that on the third day after modeling, the levels of ALB, PA, and TP in rat serum decreased in the model group; however, the levels of these proteins decreased less than those in the model group in the groups that were administered whey protein or compound protein (Figures 4(a)–4(c)). In addition, the ALB level in the high-dose compound protein group was higher than that in the whey protein group. However, on the eighth day after modeling, the difference mentioned above almost disappeared or decreased (Figures 4(d)–4(f)), but the TP level in the groups administered whey protein or compound protein was still higher than that in the model group. These results showed that modeling led to proteins in the serum promoting wound healing.

### 3.4. Evaluation of the Inflammatory Response

**Figure 5:**
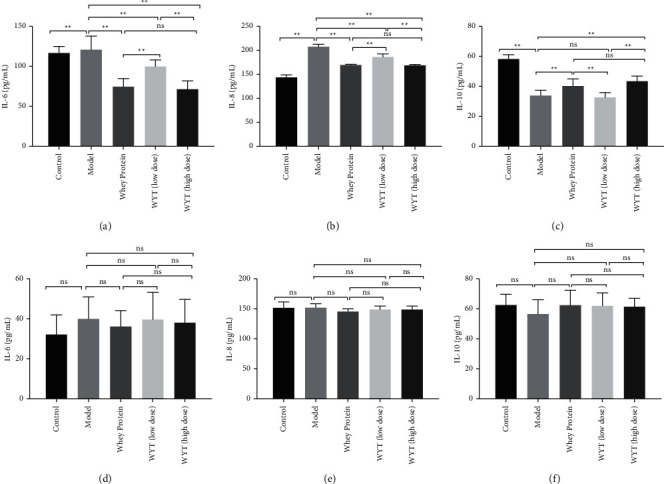
Inflammatory cytokine levels of rats on the third day (a–c) and the eighth day (d–f) after modeling. Model indicates the model group; WP indicates the whey protein group; LD indicates the low-dose compound protein group; HD indicates the high-low compound protein group. ns indicates that the significance between two groups was not significant. ^*∗*^indicates that the significance between two groups was less than 0.05 (*p* < 0.05); ^*∗∗*^indicates that the significance between two groups was less than 0.01 (*p* < 0.01).

IL-6 and IL-8 are proinflammatory cytokines, and IL-10 is an anti-inflammatory cytokine. When inflammation occurs in the body, the levels of proinflammatory cytokines increase and anti-inflammatory cytokine levels decline. In the early period after modeling (third day), the levels of IL-6 and IL-8 in rats in the model group increased and were higher than those in rats in the control group. However, IL-10 showed the opposite trend. After modeling, the IL-10 concentration declined compared to the control condition (Figures 5(a)–5(c)). However, in the groups administered whey protein or compound protein, the IL-6 and IL-8 levels were lower than those in the model group, while the IL-10 level was significantly higher than that in the model group (except for the low-dose compound protein group). This implied that the administration of whey protein or compound protein could inhibit inflammation to some extent and that the effect of high-dose compound protein was comparable to that of whey protein. Considering that the IL-6 and IL-8 levels in the high-dose compound protein group were significantly lower than those in the low-dose compound protein group and that the IL-10 level in the high-dose compound protein group was significantly higher than that in the low-dose compound protein group, the effect of the compound protein in the inflammation phase was dose-dependent. On the eighth day, the levels of cytokines returned to normal (Figures 5(d)–5(f)), indicating that the rat wounds entered the proliferation phase.

**Figure 6:**
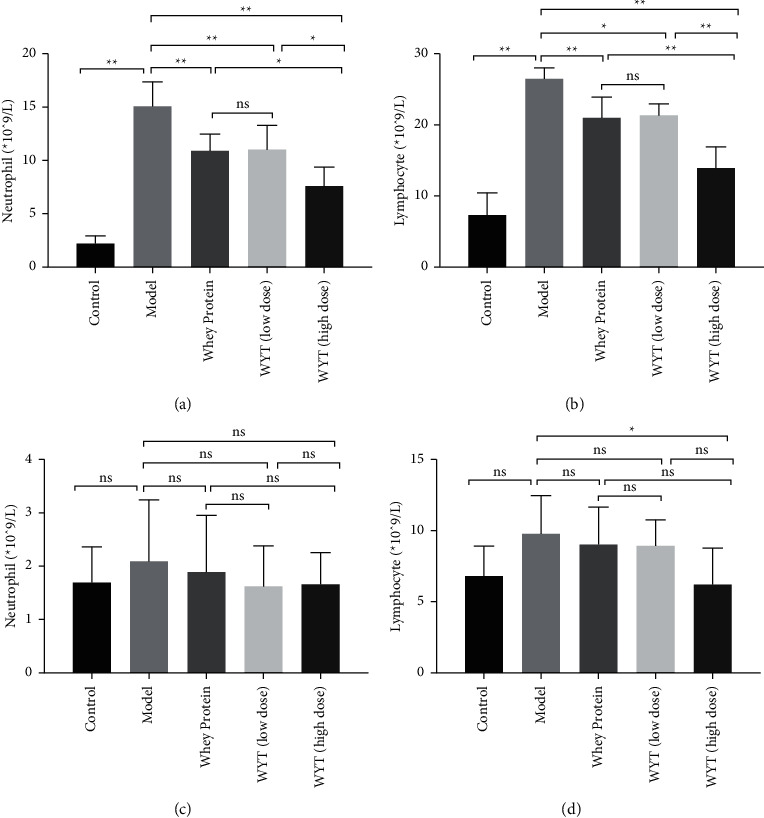
Levels of neutrophils and lymphocytes on the third day ((a, b)) and the eighth day ((c, d)) after modeling. Model indicates the model group; WP indicates the whey protein group; LD indicates the low-dose compound protein group; HD indicates the high-low compound protein group. ns indicates that the significance between the two groups was not significant. ^*∗*^indicates that the significance between two groups was less than 0.05 (*p* < 0.05); ^*∗∗*^indicates that the significance between two groups was less than 0.01 (*p* < 0.01).

Changes in neutrophil and lymphocyte counts were consistent with the trend in the inflammatory cytokine levels. As shown in Figures 6(a) and 6(b), the neutrophil and lymphocyte counts in the model and treatment groups increased and were higher than those in the control group. Among the model and treatment groups, the neutrophil and lymphocyte counts of the groups administered whey protein or compound protein were significantly lower than those of the model group, especially the whey protein and high-dose compound protein groups (*p* < 0.01). This confirmed that the administration of whey protein or compound protein could inhibit inflammation early and that the effect of compound protein was dose-dependent. Similar to the inflammatory cytokine results, most of these differences disappeared on the eighth day after modeling, and the neutrophil and lymphocyte counts returned to normal, which may be due to the disappearance of inflammation in the late period after modeling.

### 3.5. Histological Analysis

**Figure 7:**
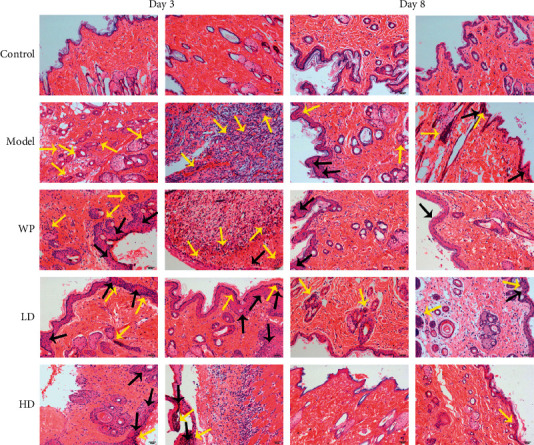
H&E staining of tissues around wounds in different groups of rats after modeling. Yellow arrows indicate where there was inflammatory cell infiltration; black arrows indicate where there was fiber proliferation. Model indicates the model group; WP indicates the whey protein group; LD indicates the low-dose compound protein group; HD indicates the high-dose compound protein group. Evaluation of IGF-1, FGF-2, and VEGF expression levels.

Figure 7 shows the pathological changes in the tissues around wounds during the wound healing process. On the third day after modeling, neutrophils (yellow arrows) appeared in the tissues around the wounds in the groups that received modeling, and there were no neutrophils in the tissue of the control group. However, fewer neutrophils were observed in the whey protein, low-dose compound protein, and high-dose compound protein groups than in the model group. Moreover, in these groups, fiber proliferation (black arrows) emerged. There was less inflammatory cell infiltration on the eighth day than on the third day in each group. In addition, the tissues around wounds in the model group and the low-dose compound protein group still had some inflammatory cell infiltration, and fiber proliferation appeared in the model group. There were few inflammatory cells in the whey protein and high-dose compound protein groups, and fiber proliferation decreased.

**Figure 8:**
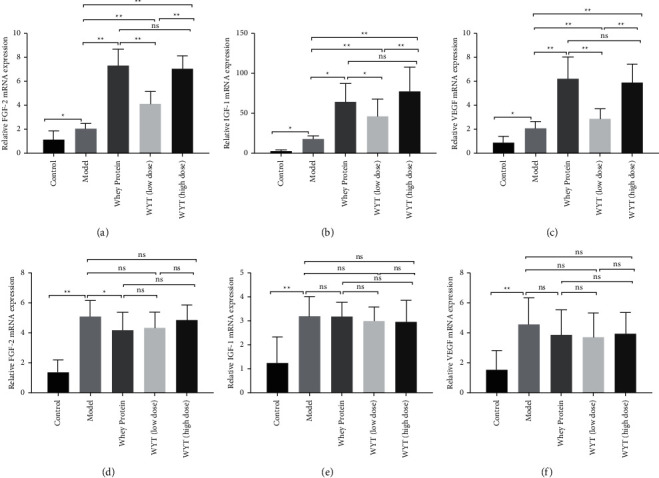
Relative IGF-1, FGF-2, and VEGF mRNA expressions on the third day (a–c) and the eighth day (d–f) after modeling. Model indicates the model group; WP indicates the whey protein group; LD indicates the low-dose compound protein group; HD indicates the high-dose compound protein group. ns indicates that the significance between two groups was not significant. ^*∗*^indicates that the significance between two groups was less than 0.05 (*p* < 0.05); ^*∗∗*^indicates that the significance between two groups was less than 0.01 (*p* < 0.01).

IGF-1, FGF-2, and VEGF are considered indicators of fibroblast proliferation, angiogenesis, and epidermization, respectively, indicating the proliferation phase in the wound healing process. These growth factors showed similar trends to those observed in the present study. On the third day, the mRNA expression of these factors increased in rats that received modeling, but in the groups administered whey or compound proteins, the mRNA expression of these factors was higher than that in the model group, except for the VEGF mRNA expression level in the low-dose compound protein group. However, these differences barely existed on the eighth day after modeling. The early expression of growth factor mRNA in the groups administered whey protein or compound protein indicated that the wounds entered the remodeling phase earlier in these groups of rats than those in the model group (Figure 8)

### 3.6. Collagen Accumulation

**Figure 9:**
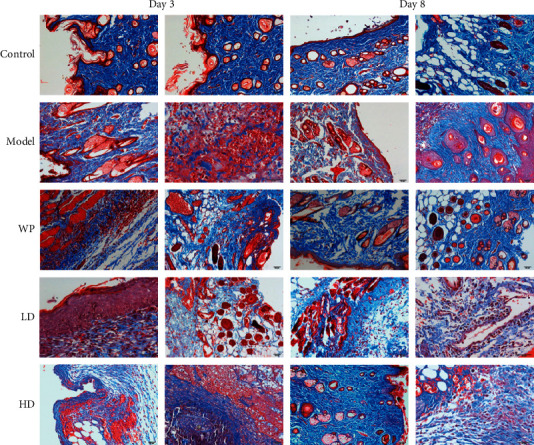
Masson staining of tissues around wounds in different groups of rats after modeling. Model indicates the model group; WP indicates the whey protein group; LD indicates the low-dose compound protein group; HD indicates the high-dose compound protein group.

In Masson staining, newly formed collagen fibers were stained blue and the muscle, red cells, and cytoplasm were stained red. In Figure 9, the blue area increased with time, indicating that the number of newly formed collagen fibers increased. On the third day after modeling, the blue area of the groups that received modeling was less than that of the control group; however, the blue areas of the high-dose compound protein and whey protein groups were larger than that of the model group. On the eighth day, the difference in the blue area between the model group and other intervention groups was more evident. Masson staining results indicated that providing whey protein or compound protein could make collagen fibers appear ahead of time in the early period after wounding.

**Figure 10:**
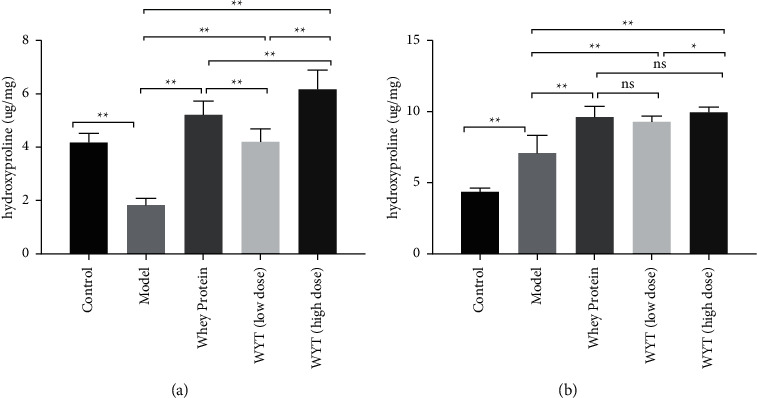
Hydroxyproline concentrations in tissues around wounds on the third day (a) and the eighth day (b) after modeling. Model indicates the model group; WP indicates the whey protein group; LD indicates the low-dose compound protein group; HD indicates the high-dose compound protein group. ns indicates that the significance between two groups was not significant. ^*∗*^indicates that the significance between two groups was less than 0.05 (*p* < 0.05); ^*∗∗*^indicates that the significance between two groups was less than 0.01 (*p* < 0.01).

We also measured hydroxyproline concentration in tissues around wounds on the third and eighth days after modeling (Figures 10(a) and 10(b)). On the third day after modeling, the hydroxyproline concentration in groups administered whey protein or compound protein was significantly higher than that in the model group (Figure 10(a)). In addition, the concentration in the high-dose compound protein group was higher than that of the whey protein group. On the eighth day, the hydroxyproline concentration increased in all groups; however, the groups administered whey protein or compound protein had higher hydroxyproline concentrations than the model group. Hydroxyproline is regarded as an indicator of collagen deposition and protein at the wound site, signifying the remodeling phase [8]. Therefore, the results demonstrated that remodeling occurred earlier in the groups administered whey protein or compound protein, and the effect of high-dose compound protein may be greater than that of whey in the early wound healing period. In addition, the compound protein's dose-response effect was observed on the third and eighth days.

## 4. Discussion

In this study, we examined the effects of oligopeptides on wound healing and nutritional status in rats. We found that providing compound proteins can effectively accelerate wound healing and improve the nutritional status of rats. Furthermore, a dose-effect relationship was observed for the compound proteins. The potential mechanism may be related to the acceleration of the entire process of wound healing, including the inflammation, proliferation, and remodeling phases.

We established a relatively objective method for calculating healing rates using a camera. In a preliminary experiment, we explored the turning points in the process of wound healing based on the results of H&E staining. The changes in H&E staining in the model groups resulted from the wound healing process, beginning with the inflammation phase, transitioning to the proliferation phase, and finally, the remodeling phase. The difference between the model and other groups was consistent with the speed of wound healing and may be due to the inhibition of inflammation by administering whey protein or compound protein, which accelerates the inflammatory phase and entry into the proliferation phase.

The experiment results (Figure 2) showed that providing compound proteins could effectively accelerate wound healing. In the plasma protein level assessment (Figure 4), changes in ALB, PA, and TP levels reflected that the uptake of compound proteins after trauma could improve nutritional status. Therefore, administering compound proteins in rats could accelerate wound healing and improve nutritional status compared to providing nothing in addition to basic energy intake in the early period of trauma. In a previous study, Akira et al. found that administering peripheral parenteral nutrition (PPN) solutions containing amino acids immediately after surgery is efficacious for wound healing [14]. Furthermore, Oishi et al. reported that protein malnutrition could deteriorate skin conditions [15]. These findings are consistent with our results, and the provision of proteins can accelerate wound healing. In addition, previous studies have shown that rapid-turnover proteins in the plasma have a short half-life and are more precise nutritional markers than albumin during the acute phase [16, 17]. In our experiment, we examined ALB, PA, and TP levels simultaneously to reflect the effect of nutritional support, and the results suggested that the compound protein can improve nutritional status in the early stages of wound healing.

The compound protein, composed of intact protein and peptides, has a lower ratio of essential to nonessential amino acids than whey protein. However, the AAS of the compound protein indicated that its amino acid pattern was similar to the WHO/FAO scoring pattern, indicating that the degree of essential amino acids being used by the body was quite high [18]. This may explain the observation (Figures 3–5) that the effect of a low dose of compound protein was inferior to that of whey protein. However, the effect of a high dose of compound protein may be equal to that of whey protein to some extent (Figures 3–5). Moreover, there was a dose-effect relationship for the compound proteins, making our results convincing. The effect of the compound protein was improved at higher doses.

Our study has several limitations. Although eight rats were sufficient to obtain meaningful results, we sacrificed half of the rats on the third day after modeling to obtain samples, leaving only four rat samples at the next time point, which may have led to bias. Therefore, in future studies, we plan to increase the sample size. Second, although we analyzed the potential mechanism in our study, it was only the manifestation of an inner mechanism. A more detailed potential mechanism involving signal transduction changes that underlie the effect of the compound protein needs to be explored. Moreover, the wound healing process is complex; thus, other factors may be involved in wound healing, and other time points may be required.

## 5. Conclusions

Our findings suggest that administering the compound protein can accelerate wound healing and improve the nutritional status. The potential mechanism may be related to the acceleration of the entire wound healing process, including the inflammation, proliferation, and remodeling phases, but the mechanisms are unclear.

## Figures and Tables

**Table 1:** Amino acid composition of the compound protein.

Amino acid	Amino acid composition of the compound protein (g/100 g)	Amino acid score (AAS) FAO/WHO 2013
Ile	3.69	1.23
Leu	6.61	1.08
Val	4.26	1.07
Lys	5.43	1.13
Met	1.30	0.90
Trp	0.66	1.00
Phe	3.36	1.19
Thr	4.14	1.66
His	1.62	1.01
Tyr	2.25	
Cystine	0.94	
Ala	5.83	
Arg	4.41	
Asp	6.41	
Glu	20.92	
Gly	8.69	
Pro	10.03	
Ser	4.41	
Essential amino acids/nonessential amino acids	0.45	

## Data Availability

The data used to support the findings of this study are available from the corresponding author (Wei Chen, email: pumchpnen@163.com) upon request.

## Additional Points

*Highlights*. (1) Administration of a compound protein can effectively accelerate wound healing and is beneficial for improving the nutritional status of rats. The dose-effect relationship of compound proteins was explored in this study. (2) The potential mechanism may be related to accelerating the entire wound healing process, including the inflammation, proliferation, and remodeling phases. (3) The high-dose compound protein treatment increased albumin and hydroxyproline levels and decreased neutrophil and lymphocyte counts compared to whey protein treatment.
